# Prevalence of left internal mammary artery disease in patients undergoing coronary angiography for suspected coronary artery disease: A meta-analysis and meta-regression study

**DOI:** 10.1016/j.ahjo.2024.100402

**Published:** 2024-05-15

**Authors:** Luca Franchin, Federico Angriman, Luca Siega Vignut, Massimo Imazio

**Affiliations:** a Department of Cardiology, Azienda Sanitaria Universitaria Friuli Centrale, 33100 Udine, Italy; b Postgraduate School of Cardiovascular Medicine University of Trieste, 34127 Trieste, Italy

**Keywords:** LIMA, CABG, Coronary angiography

## Abstract

Left internal mammary artery (LIMA) to bypass left anterior descending artery has demonstrated to improve survival in multivessel coronary artery disease, but its routine angiography during index coronary angiography is seldom performed as LIMA is rarely diseased.

A systematic literature review and meta-analysis was conducted using PubMed and Cochrane databases selecting the studies reporting prevalence of LIMA disease among patients undergoing coronary angiography and considered for CABG. Meta-regression analysis was performed to evaluate the prevalence of LIMA disease and the relative predictive value of canonical risk factors. After scrimmage, 9 studies for a total of 1365 patients were included.

LIMA disease prevalence was 1.8 % (95 % CI; 1.2 %-2.8 %) in the entire cohort, whereas we reported a rate of subclavian artery disease of 7.6 % (95 % CI, 6–9.9 %). At univariate meta-regression analysis, only age (*p* = 0.031) and smoking habit (*p* = 0.035) were directly correlated with LIMA disease.

In conclusion, LIMA angiographic assessment might be considered in selected patients that could undergo CABG, during index coronary angiography. However, prospective studies are needed to better evaluate the safety of routine selective LIMA angiography prior to CABG and whether the practice is associated with improved clinical outcomes among those individuals.

## Graphical abstract

**Figure:**
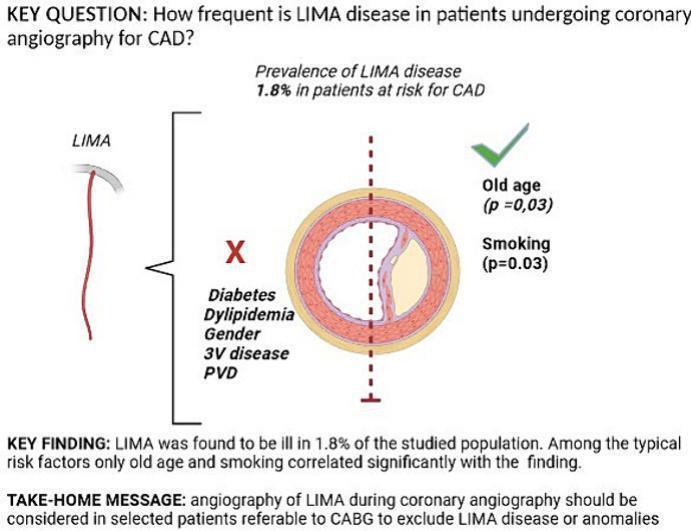

## Abbreviations

CABG: coronary artery bypass grafting
LAD: left anterior descending artery
LIMA: left internal mammary artery
PTCA: percutaneous transluminal coronary angioplasty
*PCI*: percutaneous coronary intervention

## 1 Introduction

Left internal mammary artery (LIMA) to bypass left anterior descending artery (LAD) has demonstrated to improve survival in several studies involving revascularization for multivessel coronary artery disease [[1], [2], [3]]. Compared to saphenous vein grafts, LIMA grants longer symptoms-free survival [4]. Despite the utmost importance of the vessel, it is rarely evaluated in current clinical practice before surgical revascularization. The faster way for determining the anatomy and patency of LIMA is often selective angiography during index coronary angiography. However, its routine evaluation is still debatable, especially in younger patients without many comorbidities, and when emergent or urgent revascularization preclude a thorough angiographic screening. Perspectives about when and in which patients routine LIMA angiography should be performed greatly vary between centers and operators. The reduced prevalence of diseased LIMAs, according to some studies, makes imaging of the LIMA unnecessary [5]. Some authors do not advocate routine preoperative angiography due to the risks of complications, such as dissection of the LIMA orifice, embolization with subsequent cerebrovascular ischemia, higher contrast and radiation exposure [6]. Nonetheless, they have recommended LIMA angiography for several conditions, including Takayasu's arteritis and Kawasaki disease, chest radiation history, chronic obstructive pulmonary disease and extensive aortoiliac atherosclerosis [5]. However, despite the variety of probable conditions associated with LIMA disease, data on LIMA disease prevalence are limited.

The aim of this study is to evaluate the prevalence of LIMA disease in patients undergoing coronary angiography for suspected coronary artery disease and to appraise possible predictors.

## 2 Patients and methods

The present study was performed according to the Cochrane Collaboration and PRISMA statements [[7], [8], [9]]. PROSPERO registration number is CRD42023453489.

Clinical studies that reported prevalence of LIMA disease were searched in MEDLINE/PubMed and Cochrane database (until May 2023). We restricted our searches to human populations. The search strategy included the following query: “(internal mammary artery) AND (angiography)”, as well as additional text words (such as abbreviations) in combination with an established search strategy for MEDLINE/PubMed/Cochrane database. We also hand-searched bibliographies of identified studies.

Database search and screening of duplicated results along with study selection was performed by three independent reviewers (LF, FA, LSV), with differences resolved by consensus after voting (the agreement of two out of three reviewer was necessary for the final decision). References were first scanned at the title/abstract level and subsequentially retrieved in full text. They were considered suitable for inclusion if: 1) reporting on LIMA disease prevalence, 2) including patients undergoing coronary angiography for suspected coronary artery disease. To avoid selection bias, we excluded studies that enrolled patients with the following clinical situations were excluded: a) LIMA assessed by computer tomography for other reasons, b) intraoperative surgical LIMA evaluation, c) pediatrics populations, d) post coronary artery graft by-pass populations.

Data abstraction and study appraisal were performed by three independent reviewers (LF, FA, LSV), with differences resolved by consensus. Key study and patient characteristics were extracted, including age, gender, cardiovascular risk factors, comorbidities and coronary artery disease extent.

The primary endpoint was the prevalence of LIMA disease in the analyzed cohort of patients. Diseased LIMAs were defined differently in the selected articles as LIMAs unsuitable for grafting due either to the presence of identifiable atherosclerotic lesions, vessel occlusion, excessive narrowness of the vessel, or vessel aneurism (Supplementary Table 3).

The secondary endpoints were the prevalence of left subclavian artery disease (stenosis >50 % of the lumen of the vessel), adjunctive contrast medium administration, fluoroscopy time and complication rates.

The quality of included studies was independently appraised by 3 reviewers (LF, FA, LSV), with disagreements resolved by consensus. For each included paper, we evaluated the risk of bias (low, unclear, or high) for random-sequence generation, allocation concealment, blinding of patients and physicians, incomplete outcome evaluation, and selective reporting according to the Newcastle-Ottawa scale for observational studies.

### 2.1 Statistical analysis

Continuous variables are reported as mean (SD) or median (interquartile range, IQR). Categorical variables are expressed as n (%). Statistical pooling for incidence estimates was performed according to a fixed-effect or random-effect model with generic inverse-variance weighting depending on statistical homogeneity, computing risk estimates with 95 % confidence intervals (CIs), using using RevMan 5.2 (The Cochrane Collaboration, The Nordic Cochrane Centre, Copenhagen, Denmark) and Comprehensive Meta Analysis version 3.3.070 software (Biostat, Inc. Englewood, NJ, USA). Meta-regression analysis was performed to assess the impact of baseline features on the primary endpoint. Hypothesis testing for statistical homogeneity was set at the 2-tailed 0.05 level and based on the Cochran Q test, with I2 values of 25 %, 50 %, and 75 % representing mild, moderate, and severe heterogeneity, respectively.

## 3 Results

**Fig. 1:**
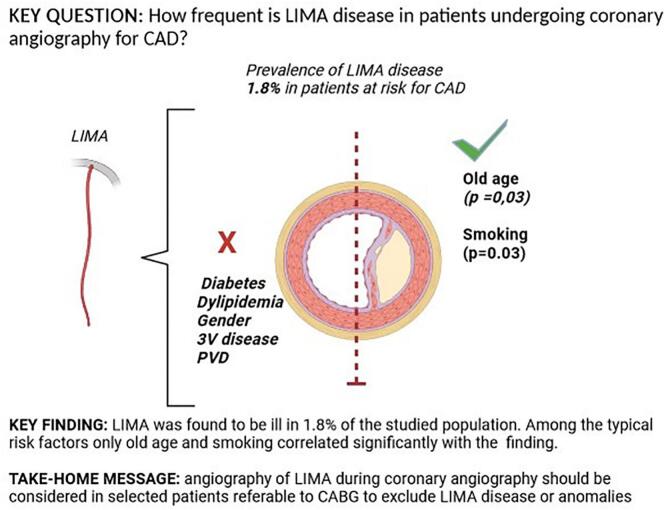
Visual abstract.

Of the 2300 studies published between 1959 and 2023, 9 studies for a total of 1365 patients were included (see Fig. 1). [[10], [11], [12], [13], [14], [15], [16], [17], [18]] The median sample size was 114 (IQR 101–150) patients with the 50 % of studied performed in Europe, the 40 % in the U.S.A and the 10 % in Asia.

In the overall cohort, the median age was 58 (IQR 57.9–64.6) years, 38.2 % were women and the 26.1 % were diabetic (see Table 1). 23.3 % had a history of previous myocardial infarction while only 5.75 % had peripheral vascular disease (see Table 1). Noteworthy about half of the cohort included patients with three-vessel disease 44.7 % (IQR 41–45.8) and the 74.2 % (IQR 44–74.6) had history of smoking (Table 2).

**Table 1:** Baseline characteristics of the population; US = United State.

First Author	Publication year	Population (n)	Study design	Area	Age (mean)	Smoking (%)	Female gender (%)	Hypertension (%)	Hyperlipemia (%)	Diabetes (%)	Previous MI (%)	PVD (%)	Trivessel disease (%)
Alcalá [12]	1997	119	Prospective	Europe	58	48	23	44	31	28	40	3	41
Bauer [13]	1990	262	Prospective	Europe	57.9	–	–	–	–	–	–	–	–
Chen [14]	2004	86	Prospective	Asia	63.60	26.12	28.26	42.07	31.84	17.81	–	–	13.95
Feit [15]	1992	130	Prospective	US	61.3	–	40	–	–	8.5	–	8.5	–
Finci [16]	1990	100	Prospective	US	56	–	–	–	–	–	–	–	–
Karabulut [17]	2012	335	Prospective	Europe	56.4	32.8	49.9	74.6	–	27.5	11.3	2.9	11.9
Krijne [10]	1990	105	Prospective	US	58	–	–	–	–	–	–	–	62
Rigatelli [11]	2005	78	Retrospective	Europe	67.8	54.2	40	74.2	70	47.8	22.8	–	49.41
Singh [18]	1983	150	Prospective	US	53.4	–	20.6	–	–	–	–	9.3	–

**Table 2:** LIMA disease and subclavian disease prevalence and outcomes.

*Study*	Population (N)	LIMA diseased (%)	Subclavian artery disease (%)	Excluded for grafting (%)	Surgical technique adaptation (%)
Alcalá	119	2,5	1,6	2,5	3,36
Bauer	262	1,2	14,1	2,7	1,50
Chen	86	1,2	5,8	ND	ND
Feit	130	2,3	2,3	4,6	1,5
Finci	100	0	ND	ND	ND
Karabulut	335	1,2	2,7	ND	ND
Krijne	105	1,0	1,9	ND	ND
Rigatelli	78	5,1	7,7	ND	ND
Singh	150	1,3	ND	ND	ND
TOTAL	1365	1,5	5,8	3,1	1,9

**Fig. 2:**
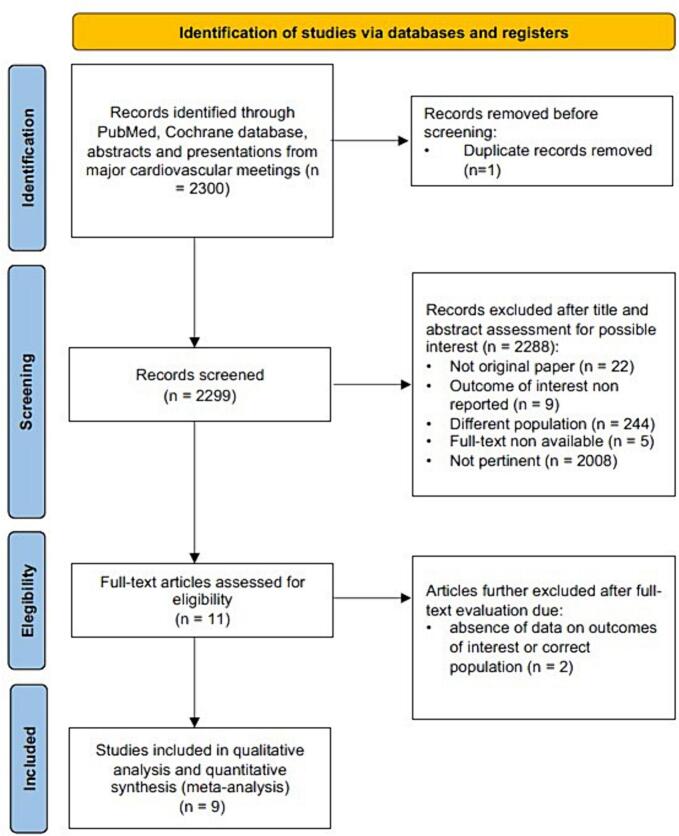
Meta-analysis PRISMA flow diagram.

As shown in Fig. 2 LIMA disease prevalence was 1.8 % (95 % CI, 1.2 % - 2.8 %) in the entire cohort. Interestingly, at univariate meta regression analysis only old age (β 0.10; 95 % CI, 0.0098 to 0.20; *p* = 0.031) and smoking habit (β 0.06; 95 % CI, 0.0041 to 0.1162; *p* = 0.035) were directly correlated with LIMA disease. On the other hand, no other risk factor or clinical characteristics correlated with LIMA involvement: female gender (β −0.0001; 95 % CI, −0.0537 to 0.0535; *p* = 0.99), hypertension (β 0.0081; 95 % CI, −0.0521 to 0.0684; *p* = 0.79), diabetes (β 0.0264; 95 % CI, −0.0111 to 0.0640; *p* = 0.16), peripheral vascular disease (β 0.0029; 95 % CI, −0.0154 to 0.0211; *p* = 0.75) and three-vessel disease (β 0.021; 95 % CI, −0.0111 to 0.0549; *p* = 0.19) (see Supplementary Table 1 and Supplementary Figs. 1–4).

**Fig. 3:**
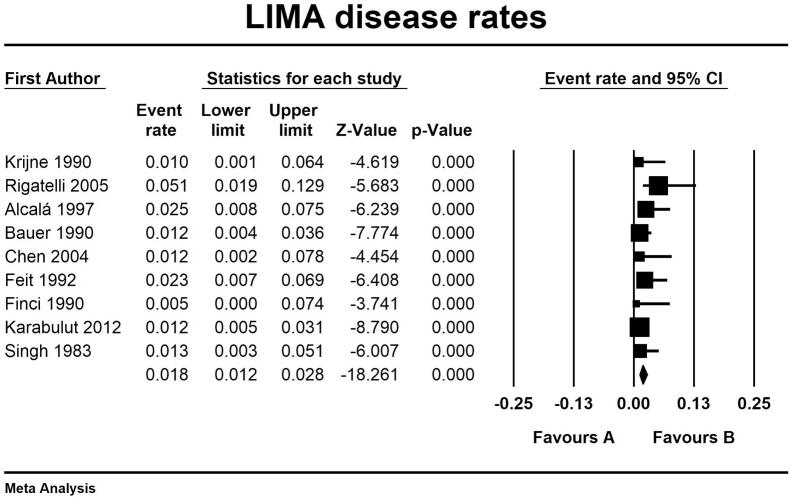
LIMA disease rates and regression analysis.

**Fig. 4:**
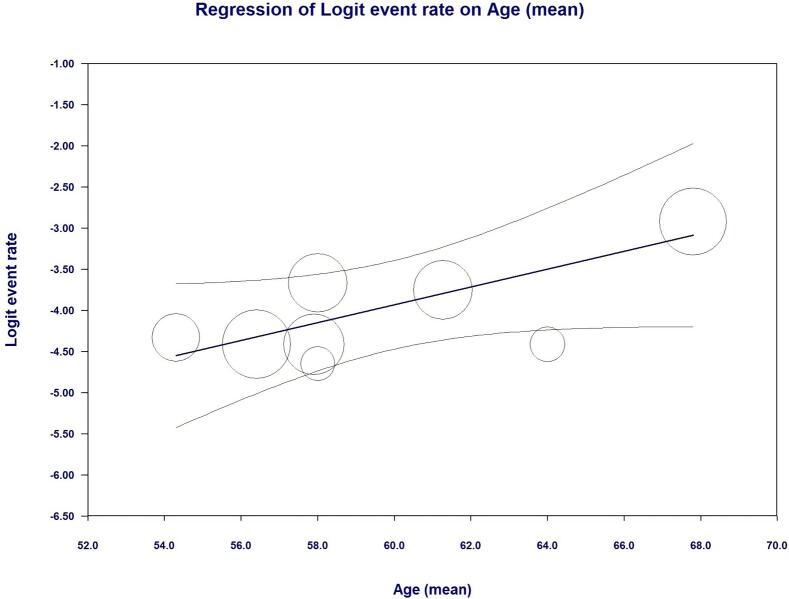
Regression analysis of LIMA disease and old age.

**Fig. 5:**
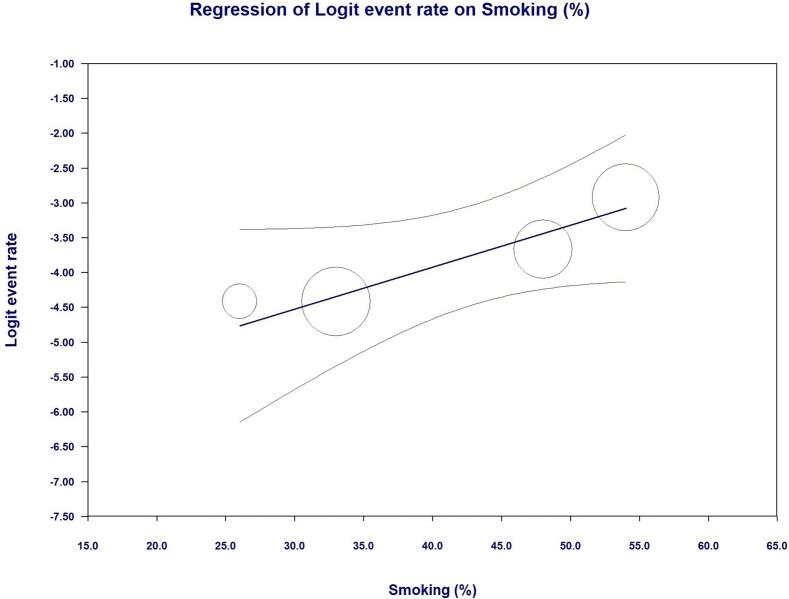
Regression analysis of LIMA disease and smoking habit.

**Fig. 6:**
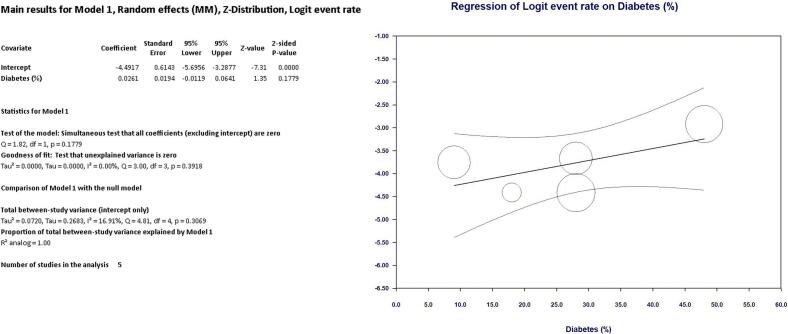
Regression on Diabetes.

**Fig. 7:**
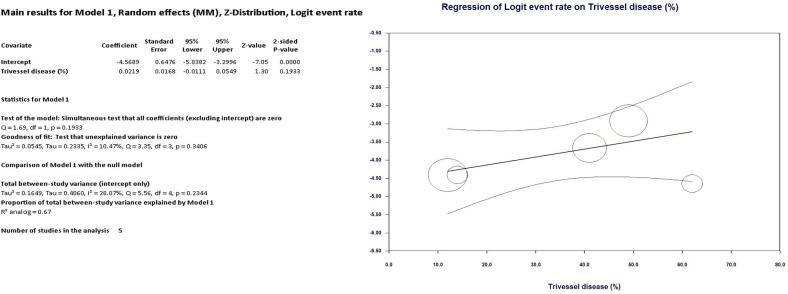
Regression on Trivessel disease.

**Fig. 8:**
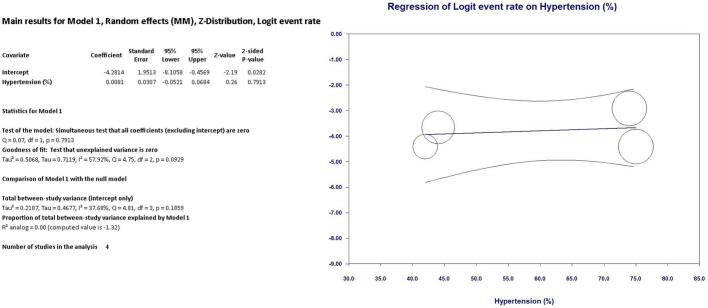
Regression on Hypertension.

**Fig. 9:**
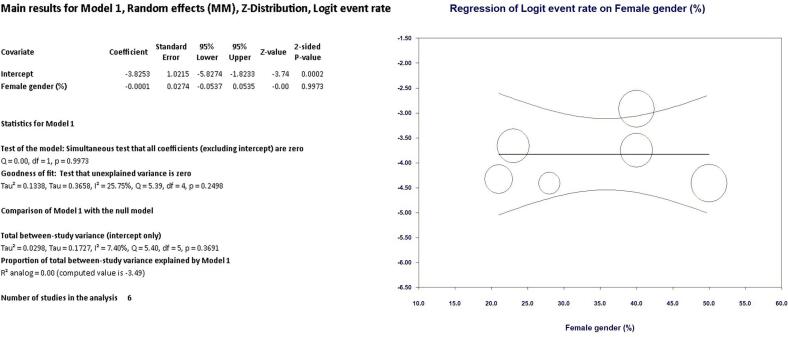
Regression on female gender.

**Fig. 10:**
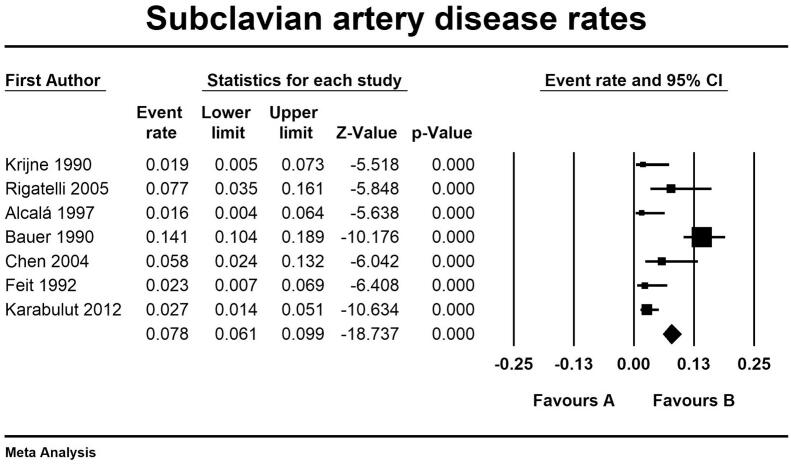
Rate of subclavian artery disease.

The secondary outcomes we evaluated were the prevalence of left subclavian artery disease and the characteristic of the LIMA selective angiography protocols. We reported a rate of subclavian artery disease of 7.6 % (95 % CI 6.1–9.9 %) (see Supplementary Table 2). As in Supplementary Table 3 the LIMA study protocols adopted required the administration on average of 11.89 ml of contrast medium and an additional fluoroscopy time of 3.27 min. A selective or semi-selective angiography approach of LIMA was obtained in 91 % of the patients and it resulted in successful visualization of the vessel in 95.38 % of the procedures; there were no reported side effects during LIMA incannulation and a single event of transient loss of visual filed during a non-selective contrast injection in the subclavian artery (Fig. 3, Fig. 4, Fig. 5, Fig. 6, Fig. 7, Fig. 8, Fig. 9, Fig. 10).

Only three studies [12,13,16] report the impact of angiography findings on clinical decision making. Among these, considering a total population of 481 patients, in 107 cases change of surgical strategy or meticulous preparation was required following the angiography findings, while in 13 cases LIMA was considered unsuitable for grafting due to atherosclerotic disease, spasm or narrowness.

Bias evaluation confirmed a moderate quality level for all studies included in this analysis (see Supplementary Table 4). Heterogeneity was generally low: for the primary and the secondary outcomes the heterogeneity statistic I^2^ was 0 %. Graphical inspection of funnel plots did not show significant asymmetries confirmed by Egger's test for the primary or secondary endpoints in any of the analyses that were performed (see Supplementary Figs. 7 and 8).

## 4 Discussion

Our meta analysis highlighted in a selected population of patients undergoing coronary angiography for suspected coronary artery disease a prevalence of LIMA disease as high as 1.8 %, as well as significant subclavian artery disease in 7.6 % of the same group. By further characterizing our population we found that among typical cardiovascular risk factors and clinical characteristics only age and smoking habit appear to directly correlate with LIMA lesions.

In the last few decades, along with the mounting evidence of increased survival for surgical revascularization with the LIMA, a few single-center studies individually inquired the condition and patency of LIMAs during coronary angiography. However, there has been no effort up to this date to collect these data to analyze the impact of LIMA disease in the management of patients in need for surgical revascularization. In our article we presented the meta analysis of 10 studies evaluating LIMAs in patients undergoing coronary angiography with the suspicion of coronary disease. The assessment of LIMA was conducted with a specific protocol of selective or sub-selective LIMA angiography, requiring extra contrast medium and extra study time compared to regular coronary angiography.

In a total population of 1365 individuals, LIMA was found to be diseased or anomalous in 1.8 % of the patients. The main abnormality consisted in atherosclerotic lesions while primary stenosis was rarer. After performing linear meta-regression analysis, we highlighted age and smoking habit as the only risk factors strongly and significantly associated to abnormal findings at LIMA angiography. Among the other population's characteristics, we took into account namely diabetes, hypertension, gender, three-vessel disease and peripheral disease, none achieved statistical significance. Moreover, the incidence of LIMAs' lesions did not clearly correlate with the number of diseased vessels at coronary angiography, even though we would care to point out a growing non-significant trend in the regression analysis with multivessel coronary disease.

Even though theoretically LIMAs share with the coronary arteries the same risk factors (old age, smoking, obesity, dyslipidemia), possibly leading to intimal and wall degeneration, our data do not support a clear correlation with most of these. Also, the prevalence of disease appears to be significantly lower compared with other arterial districts: in confirmation of our 1.8 % of diseased vessels at angiography, old data based on autopsies report rates of atherosclerotic disease of LIMA between 3.1 and 4.2 % in the general population [19] and 0.7–7 % in patients with multivessel disease [20].

Based on these assumptions and the presented date, we believe that the characterization of LIMA anatomy and flow ahead of coronary artery bypass grafting (CABG) could be of significant value in preventing late changes in surgical techniques as well as early failure of the graft. The procedure itself required on average a low quantity of contrast medium (11,9 ml) and few minutes to be performed (3.2 min) and proved to be safe with only one complication reported during a non-selective subclavian injection (transient partial loss of sight). Lately, CABG has been accepted as a safe and reliable solution for coronary obstruction in the context of a diffuse coronary disease or difficult coronary anatomies. A great reduction in short-term mortality rates in the last three decades as well as significant benefits compared to PTCA in multivessel patients (particularly in the diabetic population) paved the way towards a surgical first-line approach in selected patients [21,22]. However, to guarantee an efficient and lasting result, surgery still relies on the use of homologous vessels, mainly the LIMA and saphenous veins, with rare use of radial artery grafts. Of course, this technique relies on the assumption that the graft conduit is pervious and healthy to grant a durable blood flow to the myocardium. Therefore, even though the rate of diseased vessel is low, selective angiography of LIMA, being a fast, safe and cheap procedure, could still be a cost-effective evaluation in selected patients expected to undergo CABG.

### 4.1 Limitations

Our meta-analysis has different limitations. First, only observational studies were included in our analysis with considerable variation in type of demographic characteristic as well as limited representation of women (only 38.3 %) and minorities (data on that concern were not available). Also, definition of coronary artery disease extension and sample sizes among the studies were significantly different. Secondly, despite a low statistical heterogeneity between studies, different modalities of LIMA visualization were used across studies such as selective or unselective injections of the vessel. Thirdly, the definition of LIMA disease was not perfectly consistent among the studies. Finally, because of the lack of raw data, we could not draw conclusions about the specific subgroup of patients who proceeded to CABG or about a clear age cut-off fir increased risk of LIMA disease. Furthermore, the studies included in the meta-analysis are quite dated (only one study was published after 2005) and therefore precede widespread adoption of radial artery access. Balaban et al. [23] found that femoral access is related to less fluoroscopy time, so LIMA visualization in the current practice may take longer time, but they also found that LIMA was best seen via the left radial route [23]

### 4.2 Conclusion

LIMA was found to be diseased in 1.8 % of the population undergoing coronary angiography is the observed cohort. No significant correlation was found between LIMA disease and classic risk factors for atherosclerosis, exception made for old age and smoking habit. Due to the low level of predictability of the disease, the ease of the procedure and its relative safety, LIMA angiographic assessment might be considered in selected patients candidate to CABG at older age and/or with smoking habit history. However, prospective studies are needed to better evaluate the safety of routine selective LIMA angiography prior to CABG and whether the practice is associated with improved clinical outcomes among those individuals.

## Funding statement

None.

## CRediT authorship contribution statement

**Luca Franchin:** Writing – review & editing, Writing – original draft, Visualization, Validation, Supervision, Software, Resources, Project administration, Methodology, Investigation, Funding acquisition, Formal analysis, Data curation, Conceptualization. **Federico Angriman:** Writing – review & editing, Writing – original draft, Visualization, Validation, Software, Resources, Project administration, Methodology, Investigation, Funding acquisition, Data curation, Conceptualization. **Luca Siega Vignut:** Writing – review & editing, Writing – original draft, Visualization, Validation, Software, Resources, Project administration, Methodology, Investigation, Funding acquisition, Data curation, Conceptualization. **Massimo Imazio:** Supervision.

## Declaration of competing interest

The authors declare that they have no known competing financial interests or personal relationships that could have appeared to influence the work reported in this paper.

## Data Availability

The data underlying this article are available in the article and in its online supplementary material.

## Appendix A Supplementary data

Supplementary tables Image 1

Supplementary figures Image 2
