# Supplementary material for: Prevalence of left internal mammary artery disease in patients undergoing coronary angiography for suspected coronary artery disease: A meta-analysis and meta-regression study
Source: Am Heart J Plus. 2024 May 15;43:100402. doi: 10.1016/j.ahjo.2024.100402 (PMC11127098; doi:10.1016/j.ahjo.2024.100402)
Supplement: Supplementary file 1 — Supplementary tables [file mmc1.docx]

**Supplementary tables**

| ***Study*** | **Population (N)** | **Selective or semi- incannulation (%)** | **Satisfactory visualization (%)** | **Additional contrast medium (ml)** | **Additional study time (minutes)** |
| --- | --- | --- | --- | --- | --- |
| **Alcalá** | 119 | 89,9 | 98,3 | **ND** | 2,8 |
| **Bauer** | 262 | 100 | 88 | 12,5 | **ND** |
| **Chen** | 86 | 100 | 100 | 5 | 3 |
| **Feit** | 130 | 93 | 93 | **ND** | 5 |
| **Finci** | 100 | 0 | 97 | 27,9 | **ND** |
| **Karabulut** | 335 | 100 | 96,4 | **ND** | **ND** |
| **Krijne** | 105 | 100 | 100 | 10 | **ND** |
| **Rigatelli** | 78 | 100 | **ND** | 5 | 1,4 |
| **Singh** | 150 | 100 | **ND** | **ND** | **ND** |
| **TOTAL** | 1365 | 91,12820513 | 94,93201407 | 11,08240223 | 1,724648787 |

Supplementary table 1 Procedural specifics

| ***LIMA ANOMALIES*** | **Prevalence (%)** |
| --- | --- |
| **Stenosis (%)** | 1,2 |
| **Obclusion (%)** | 0,8 |
| **Narrow (%)** | 1,2 |
| **Spastic (%)** | *0,4* |
| **Anomalous course (%)** | *0,4* |
| **Large branches (%)** | *8,2* |
| **Common origin (%)** | 12 |

Supplementary table 2 LIMA anomalies
