# Supplementary material for: Prevalence of left internal mammary artery disease in patients undergoing coronary angiography for suspected coronary artery disease: A meta-analysis and meta-regression study
Source: Am Heart J Plus. 2024 May 15;43:100402. doi: 10.1016/j.ahjo.2024.100402 (PMC11127098; doi:10.1016/j.ahjo.2024.100402)
Supplement: Supplementary file 2 — Supplementary figures [file mmc2.docx]

**Supplementary figures**

Supplementary figure 1 Regression on Diabetes


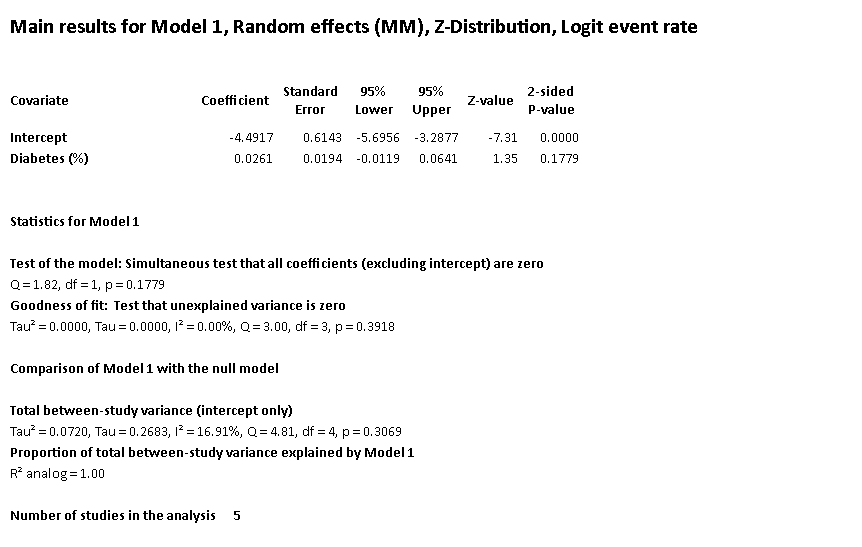


Supplementary figure 2 Regression on Trivessel disease


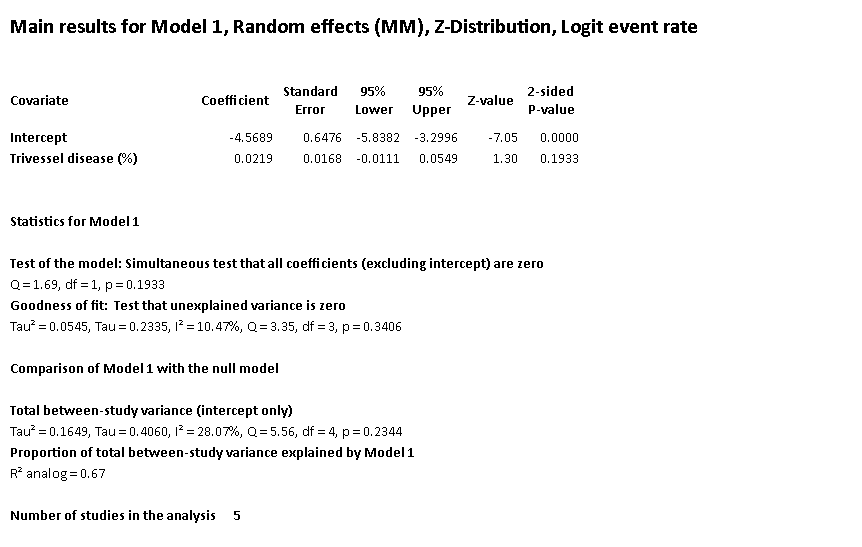

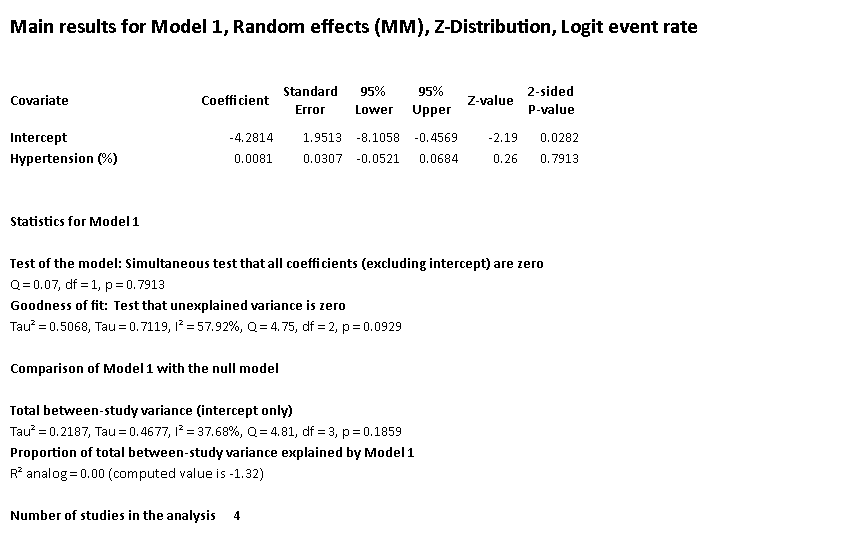


Supplementary figure 3 Regression on Hypertension


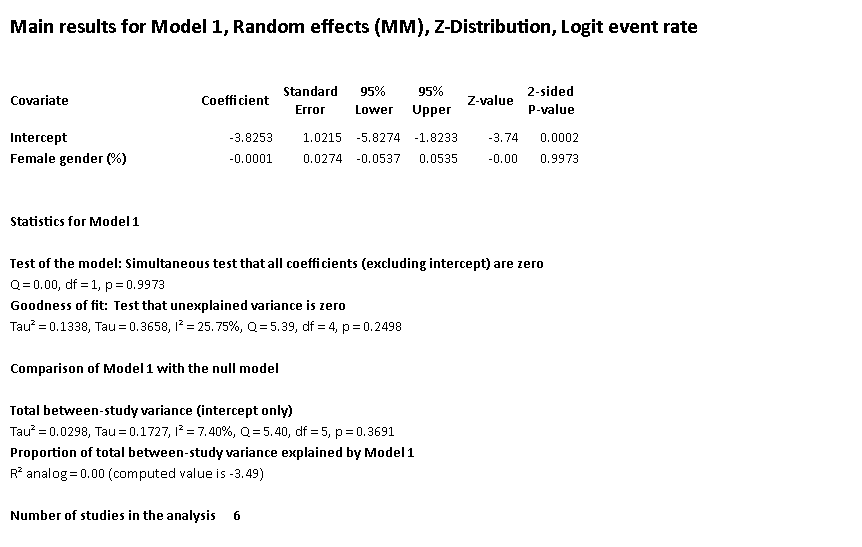


Supplementary figure 4 Regression on gender

Supplementary figure 5 Rate of subclavian artery disease
